# Behaviour of Soil–Steel Composite Bridges under Strong Seismic Excitation with Various Boundary Conditions

**DOI:** 10.3390/ma16020650

**Published:** 2023-01-09

**Authors:** Tomasz Maleska, Damian Beben

**Affiliations:** Faculty of Civil Engineering and Architecture, Opole University of Technology, 45-758 Opole, Poland

**Keywords:** soil–steel bridge, seismic analysis, nonlinear numerical analysis, boundary conditions

## Abstract

Soil–steel composite bridges typically range from 3 to 32 m, and they can be applied as an effective alternative for reinforced concrete bridges with short spans. They are able to meet the same design and safety requirements as traditional bridges more rapidly and at a lower cost. The behaviour of such bridges under seismic events is not yet recognized, because seismic excitations are completely different from the static and dynamic loads that have been analysed so far. This paper presents the results of the numerical study of two various types of soil–steel composite bridges under strong seismic excitation. The first soil–steel composite bridge has a span of 17.67 m and a height of 6.05 m, and the second consists of two shells with a span of 4.4 m each and a height of 2.8 m. Numerical analysis was performed for three models for each bridge, taking into account different boundary conditions. The applied boundary conditions are intended to represent the commonly used reinforcements of this type of bridges (reinforced concrete collar, reinforced concrete front wall). The obtained results were compared with the model in which such reinforcements were not used. Calculations were conducted using the DIANA program based on a finite element method. The non-linear models with seismic excitation of El Centro from 1940 and Time History analysis were applied. The conclusions from the study can be useful in making a decision regarding the design of the soil–steel composite bridges located in seismic zones. In addition, it was found that the effect of the applied strengthening is significant in the behaviour of soil–steel composite bridges.

## 1. Introduction

**Figure 1:**
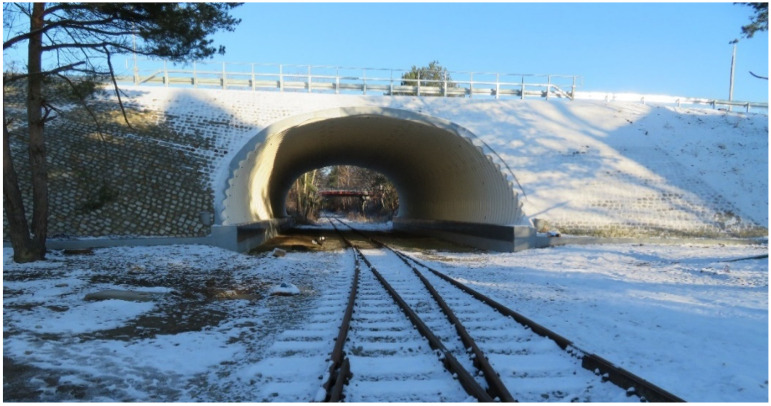
Example of soil–steel composite bridge located in Opole (Poland).

Engineering structures made of corrugated sheets (Figure 1) cooperating with the soil have been used for over 100 years. These structures are usually called soil–steel composite bridge. Elements of corrugated sheets are utilized in bridges, culverts, viaducts, tunnels, supports in opencast mines, etc. The largest number of engineering structures of this type can be found in the United States, Canada, Western Europe, and Scandinavian countries. In Poland, the first investments in this technology date back to the late 1980s [1,2,3].

The most important reasons for the dynamic increase in the number of soil–steel composite bridges include: (i) low costs of building, (ii) short period needed for construction, (iii) a minimum amount of construction material (steel) in relation to the span of the shell structure, (iv) the possibility of building shells off-site (prefabrication of the steel shell), (v) the possibility of building them throughout the year, (vi) a continuous process of construction, without the need to close traffic, (vii) no or minimal negative impact on the environment, (viii) architectural values, and (ix) compliance with the idea of sustainable development. One of the significant advantages of this type of bridges is related to soil compaction, which has a positive effect on increasing the stiffness of the structure over time. The effect of this phenomenon is the ability to carry ever greater live loads. This is a characteristic feature of these structures and is in contrast to traditional bridges, which tend to reduce their load-bearing capacity over time. In addition, soil–steel bridges, thanks to the corrugated profile of the shell structure and well-compacted backfill, are able to create a composite structure (soil–steel). In addition, due to the flexibility of the corrugated steel shell and its interaction with the backfill, these bridges are able to transfer to a large external loading. This is the opposite behaviour to traditional steel or concrete bridges.

The technology used for construction of soil–steel composite bridges made of corrugated steel plates (CSP) consists in close interaction of steel shell with surrounding soil and using the effect of arching. This phenomenon is familiar in soil mechanics, but in the analysis of soil–steel bridges this aspect plays a significant role. The aim of corrugation is to increase stiffness of the system structure and to cause interaction of the shell with backfill. Structures are composed of profiled corrugated plates, also called shells, or construction elements which were put together by means of high-strength bolts in cross-section and along the structure length. Such solutions allow for easy, quick, and economical mounting of the structure. In addition, the steel shell is protected against corrosion by hot-dip galvanizing with a thickness of 85 μm and, in the case of an aggressive environment, it should be additionally painted with epoxy paint (200 μm). These layers allow for long and safe use of these structures [1,2].

**Figure 2:**
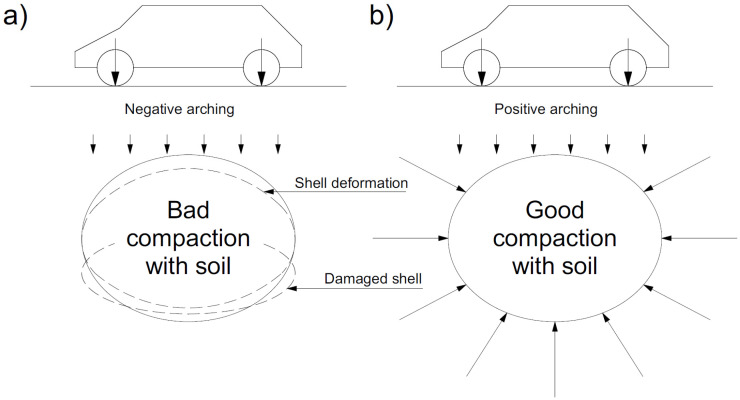
Idea of soil–steel bridge with arching phenomenon: (**a**) negative and (**b**) positive.

Another important element in soil–steel composite bridges is the arching phenomenon in the soil. The arching factor is a phenomenon of load redistribution on the shell as a result of the formation of shear stresses that oppose displacements in the soil medium. Vertical loads tend to reduce the height of the shell and at the same time increase its span (negative arching—Figure 2a). On the other hand, the displacement of the shell in the horizontal direction causes positive arching (Figure 2b), which reduces the possibility of increasing the span, and thus the height of the shell [1,2,4].

The analysis involving soil–steel composite bridges under static load focuses primarily around the search for an answer to the question: what will be the deformation of the steel shell caused by laying and compaction of the backfill (at the construction stage) [5,6,7,8,9,10,11,12,13]. In the static loads, the main research area for soil–steel bridges has been their response to trucks positioned statically on the bridge [14,15,16,17,18,19,20,21,22]. Based on the test results, it can be observed that the maximal displacements were smaller than in the backfilling process.

In the case of dynamic loads, the main goal of each paper was to perform the dynamic identification of these structures (modal analysis) and their response to variable loads (due to vehicle traffic) [17,23,24,25,26,27,28,29,30,31,32,33,34]. In addition, the dynamic loads, which are difficult to predict due to their random characteristics, should also be taken into account. Such dynamic loads include seismic loads caused by an earthquake or anthropogenic loads caused by human activity, e.g., mining tremors. So far, the finite element method has been used in the analysis of the impact of seismic tremors on underground soil–steel composite structures. As part of the numerical tests, the influence of the soil cover height above the steel shell [35], the influence of stiffening elements [36], and the influence of the EPS layer damping seismic and anthropogenic excitation [37] were determined. In addition, research works [38,39] attempted to determine the impact of seismic shocks based on the observed damage caused by seismic excitation. It was noted in works [40,41,42] that the impact of seismic tremors on soil–steel objects is significant, and numerical analysis can be used to assess their behaviour under seismic inputs. 

The main aim of the study was to establish a reply to the following question: what influence does seismic excitation have on the safety of soil–steel composite bridges under given boundary conditions? The paper reports the results of numerical analysis of soil–steel bridges subjected to strong seismic loading for various designs of bridge cross-section. Therefore, two soil–steel bridges were used in the study. The numerical program DIANA FEA was used for the numerical analysis. The seismic record from the El Centro earthquake was applied as the source of strong seismic excitation. The conducted research will be helpful in designing this type of object located in seismic areas. 

## 2. Methodology

### 2.1. Description of the Real Bridges

Two soil–steel bridges made of corrugated sheets were analysed in this paper. The tested objects are located in Poland, where there are no significant natural seismic phenomena. On the other hand, in Poland there are human induced seismic events, i.e., mining tremors, which are similar to earthquakes, but their strength is smaller. In addition, analyses of soil–steel bridges and culverts under seismic load are relevant for countries where seismic phenomena occur frequently, and the conclusions of this work can be used to assess the behaviour of this type of bridge. 

In order to assess the seismic behaviour of soil–steel composite bridge with various structural reinforcements (RC collars, RC front walls), two different types of such bridges were selected. The first bridge has an open steel shell structure founded on the RC footings, and the second one has a closed profile founded directly on the ground. It should be mentioned that both bridges were previously tested under static [12] and dynamic loads [28,32]. The detailed characteristics of the tested soil–steel bridges are presented below.

#### 2.1.1. Bridge #I with Single Steel Shell

**Figure 3:**
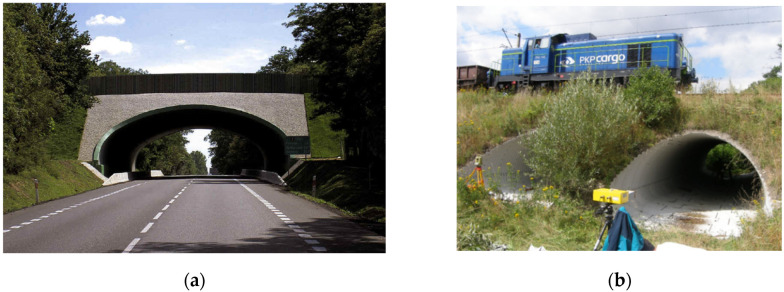
Side view on the soil–steel composite bridges used for numerical analysis: (**a**) single shell located in Trzebaw (Poland), and (**b**) double shell located in Krosnowice (Poland).

**Figure 4:**
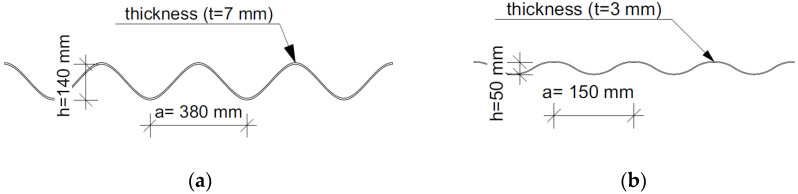
Dimensions of corrugated steel plates of bridge: (**a**) #I and (**b**) #II.

**Figure 5:**
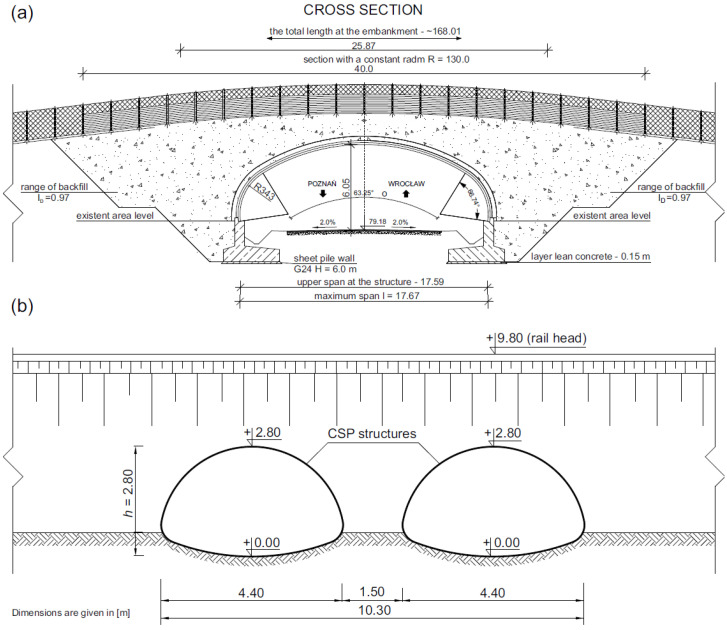
Cross section of: (**a**) bridge #I with single steel shell, (**b**) bridge #II with double steel shell.

**Figure 6:**
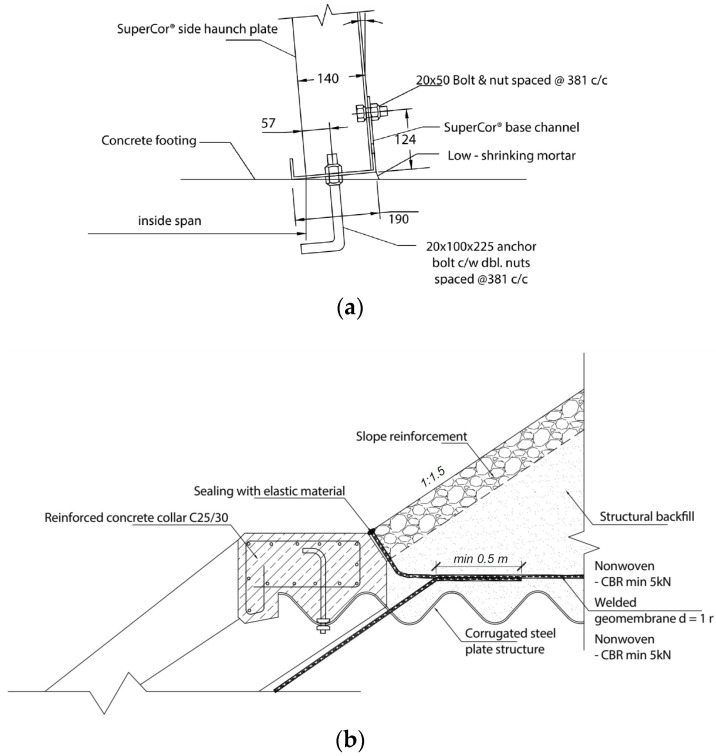
Example of: (**a**) footing connection with CSP shell and (**b**) RC collar connection with CSP shell.

The first of the analysed structures was a soil–steel composite bridge, also known as the ecological viaduct (ecoduct—Figure 3a) and was built of corrugated plates with the so-called deep wave. The object was located in Trzebaw within the Wielkopolski National Park, on the national road no. 5 between Wroclaw and Poznan. The object in the longitudinal section was a structure in the form of a single-span thin-walled shell made of CSPs with dimensions of 140 × 380 mm and a plate thickness of *t* = 7 mm (Figure 4a). Taking into account the corrugation parameters, the area cross-section was 8.87 mm^2^/mm and the moment of inertia was 21,897.45 mm^4^/mm. A steel shell with a span of 17.67 m (Figure 5a) was fixed in the strip reinforced concrete (RC) footings by means of steel C-profiles and then anchored (Figure 6a). The span width in the shell crown was 40.39 m, while at the foundation it was 55.83 m. In the plane, the structure was located perpendicular to the road. Two massive RC footings were 4.0 m wide at the base and 57.83 m long. They were made of C30/37 class concrete. The height clearance of the structure was 6.05 m (Figure 5a). The individual CSPs were connected to each other by means of M20 class 8.8 high-strength bolts. Both ends of the shell structure on the inlet and outlet sides were secured and strengthened by forming a RC collar with dimensions of 0.4 × 0.6 m (Figure 6b).

The structure of the shell made of CSPs was covered with layers of soil (about 0.2 m thick). These layers were then properly compacted according to the Proctor scale to 0.95 for the backfill in direct contact with the steel structure and 0.7 for the remaining part of the backfill [12]. Above the steel shell crown, the height of the backfill was 1.8 m (Figure 5a). Such backfill height also provides adequate damping of the vibrations in the shell structure and reduces the noise level resulting from vehicular traffic. Moreover, such implementation of backfill layers made it possible to plant trees and shrubs and grow other vegetation. The inlet and outlet of the analysed structure were finished in the form of slopes lined with stone cubes (Figure 3a). This type of finishing is intended to properly integrate the facility into the surrounding natural environment of the Wielkopolski National Park.

#### 2.1.2. Bridge #II with Double Steel Shell

The second analysed object is a railway soil–steel composite bridge, which consists of two shells (Figure 3b). These shells are characterized by a pipe-arch cross-section and are constructed of CSPs with dimensions of 50 × 150 mm and a thickness of *t* = 3 mm (Figure 4b). Taking into account the corrugation parameters, the area cross-section was 3.77 mm^2^/mm and the moment of inertia was 1176.60 mm^4^/mm. The facility is located in Krosnowice in Dolnośląskie Province. The shells in the bridge structure have a span of *L*_1_ *= L*_2_ *=* 4.4 m and are erected directly on the ground (Figure 5b). The backfill was laid in layers of about 0.2–0.3 m and compacted according to the Proctor scale to 0.95–0.98. The clear height of the shells was 2.8 m. The individual sheets of the CSPs were fixed with M20 high-strength bolts. The height of the backfill above the steel shells was equal to 2.4 m. The length of the bridge in the steel shell crown was 16.0 m and at the base 21.8 m. In this bridge, no additional elements were applied to strengthen the steel shells, e.g., in the form of reinforcement ribs. It can be added that from the inlet and outlet sides, the RC collars were built at the ends of the steel shell, just as in the case in the bridge located in Trzebaw. In addition, the walls of the inlet and outlet were covered with grass without additional reinforcements in the form of paving stones, etc. (Figure 3b).

### 2.2. Material Properties of Numerical Model

**Figure 7:**
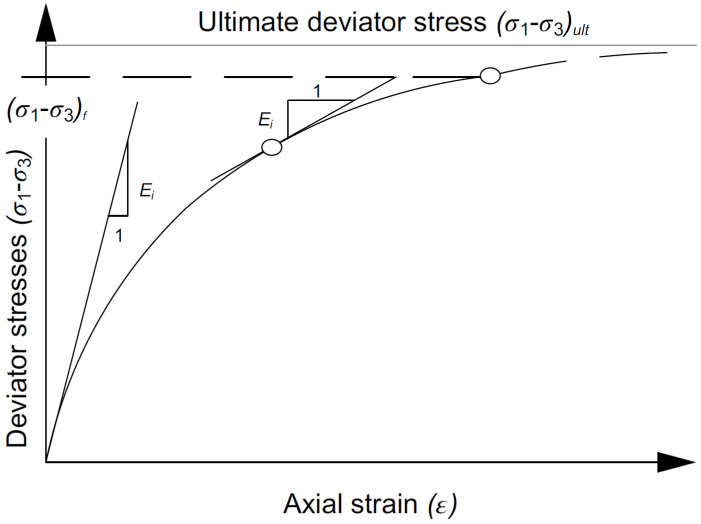
Duncan–Chang nonlinear hyperbolic constitutive model of soil for bridge #I and #II.

This study applied the following sources for material characteristics: the manufacturer data (for the steel shell), research and parametric analysis in the numerical analysis, and insights gained from the literature review: (i) backfill (Figure 7) [43,44] and data obtained from ViaCon Poland, (ii) interface layer [45], and (iii) boundary conditions [38,40,41,42,46,47,48,49]. All material characteristics applied in the analysis are summarized in Table 1. It should be noted that the corrugation shells of the bridge were modelled as complex structures without applying any simplifications or transformations. In addition, the damping ratio of dynamic analysis was equal to 5%, which is in agreement with [50,51].

**Figure 8:**
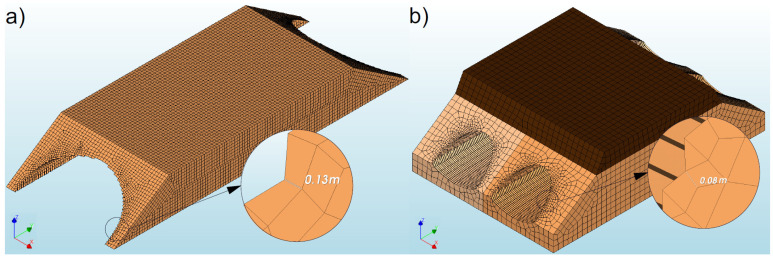
View of FEM models of the analysed bridges: (**a**) #1, (**b**) #2.

The size of the finite elements used to model the analysed bridges (#I and #II) did not exceed 0.5 m. It should be added that this is the largest assumed size of the finite element, which corresponded to the soil element, e.g., in the top and sides of the numerical models (Figure 8). In addition, the mesh of finite elements with the densest division was located near the shell, i.e., at the junction of two materials (soil and steel). In these places, the size of the finite element was only a few centimetres. In the case of bridge #I, 105,775 finite elements were used, and 108,204 elements were used for the discretization of bridge #II. Thus, the models from the conducted analyses were as close as possible to each other, and subsequent densities of the mesh did not increase the discrepancy of the results.

### 2.3. Boundary Conditions

#### 2.3.1. General Remarks

In order to adequately represent the performance of the structure, it is also very important to adequately define the boundary conditions for the analysed numerical models [1,2,40,41,46,47,48,49]. Pinned supports were used as the boundary conditions for the soil (model base) and the shell. The longitudinal walls of the numerical model were also modelled as pinned supports, similar to the recommendation from the papers [40,41,52,53,54]. Pinned supports were used because this type ensures a continuum of seismic wave propagation without its reflection from the edges of the numerical model, which is important in soil–steel bridges due to the composite structure.

#### 2.3.2. Description of Boundary Conditions in Numerical Analysis

**Figure 9:**
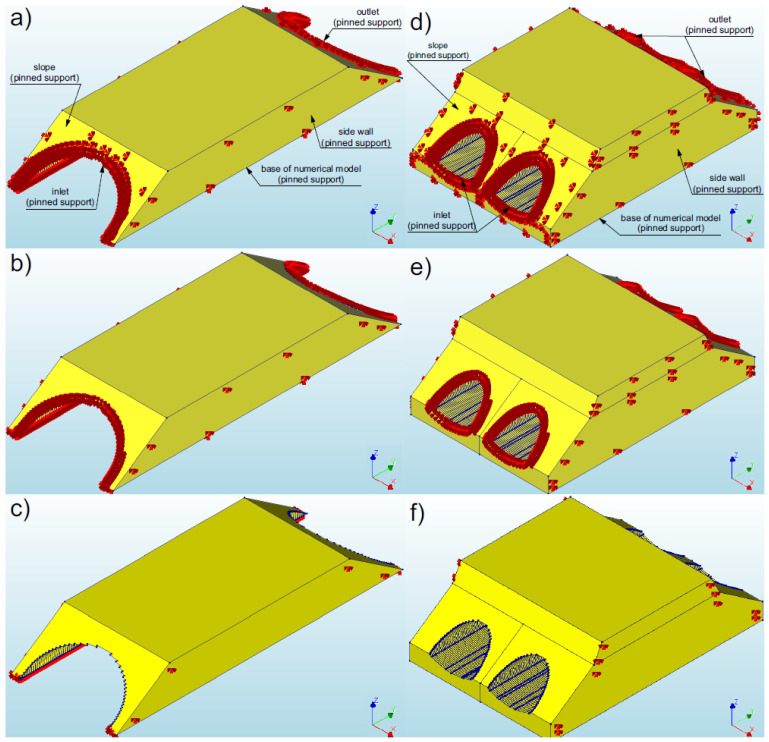
View of the boundary conditions for bridge #I: (**a**) model 1, (**b**) model 2, (**c**) model 3 and for bridge #II: (**d**) model 4, (**e**) model 5, (**f**) model 6.

Three numerical models were applied in the analysis of the effect of the boundary conditions (various types of reinforcements) on the response of soil–steel composite bridges subjected to seismic excitation for each structure (models 1–3 (bridge #I) and models 4–6 (bridge #II)):

- Model 1 and 4: For the bridge slopes, inlet, outlet (shell), and the base of the numerical model together with the side walls were modelled as pinned supports (Figure 9a,d). This case was intended to reproduce the use of a RC collar acting as a reinforcement of the shell (at its inlet and outlet) and the RC front wall (on slopes), as well as the execution of the object in an excavation.
- Model 2 and 5: The inlet and outlet as well as the base of the model (shell and soil) together with the side walls were modelled as pinned supports (Figure 9b,e). This case was designed to simulate the use application the RC collars at the ends of the shell, and only grass planted on the slopes (instead of RC front wall), as well as the execution of the object in an excavation,
- Model 3 and 6: The base of the model (shell and soil) together with the side walls were modelled as pinned supports (Figure 9c,f). This case was to determine the effect of a RC collar that was applied to stiffen the shell (i.e., comparison of models 2 and 3).

### 2.4. Interface

The interface between the two materials (steel and soil) forms one of the most important elements that were applied in soil–steel composite bridges and one that has a significant effect on their load capacity. For this reason, for the adequate description of the soil–steel bridge model, it was necessary to apply a specific approach so that interface zones and boundary conditions are defined adequately. The parameters of the contact layer (the so-called interface) were adapted to a specific case, i.e., to the characteristics of the soil medium (usually expressed by cohesion, internal friction angle, and soil moisture), the condition of the interface surface (roughness), the stiffness of the front walls of the object, and the duration of variable loads.

**Figure 10:**
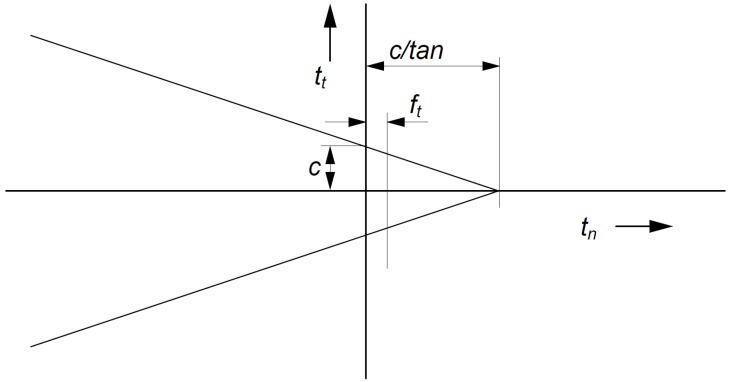
The concept of the Coulomb friction model.

The impact of the interface layer on the results obtained depends primarily on its stiffness, as well as the dimension of the object and the material from which the structure was made. The inadequate determination of these parameters may lead to unrealistic interface between these two media and, consequently, an incorrect response of the entire structure. In this paper, the following interface parameters were adopted (i) Coulomb friction model (Figure 10), (ii) rigidity of 100,000 kN/m^3^, (iii) cohesion of 3 × 10^−3^ MPa, (iv) angle of internal friction of 39°, and (v) dilation angle of 5° [45,52,53,54].

### 2.5. Seismic Excitation

**Figure 11:**
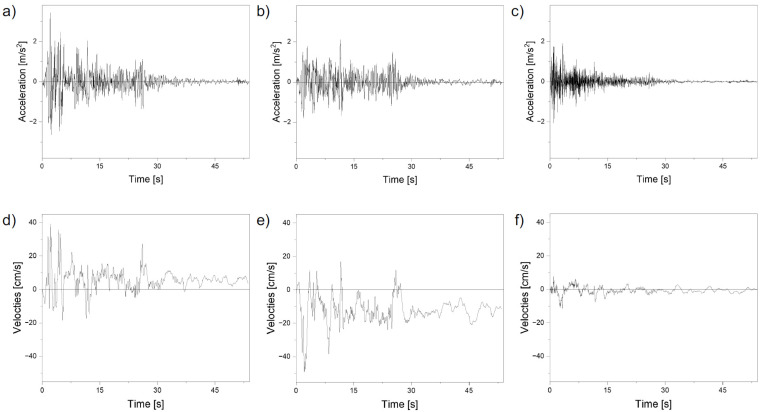
Seismic records from El Centro 1940 on the direction of accelerations: (**a**) X-NS, (**b**) Y-EW, (**c**) Z-UP; and velocities of (**d**) X-NS, (**e**) Y-EW, (**f**) Z-UP.

The Time History method, i.e., a step-by-step analysis in the time domain, was applied in the analysis of the effect of seismic excitation on the nonlinear behaviour of soil–steel composite bridges. The El Centro earthquake from the USA was adopted as the model of the seismic excitation (Figure 11). This entry dates from 18 May 1940 and is considered catastrophic. It is characterized by the intensity scale X on the Mercalli modified scale (6.9 on the Richter scale) [35,36].

## 3. Results and Discussion from Numerical Simulation

### 3.1. General Remarks

This study applied Time History analysis, with the time step equal to 0.02 s. Moreover, in the seismic analysis, the XII natural vibration mode was adopted, as it conforms with the requirements of [50] for building structures. The set seismic load was applied simultaneously in three directions, i.e., horizontal “X” and “Y” and vertical “Z”. The effect of the earthquakes from numerical analysis was presented in terms of displacements, stresses, axial forces, bending moments, velocities, and accelerations. 

### 3.2. Displacements

#### 3.2.1. Bridge #I

**Figure 12:**
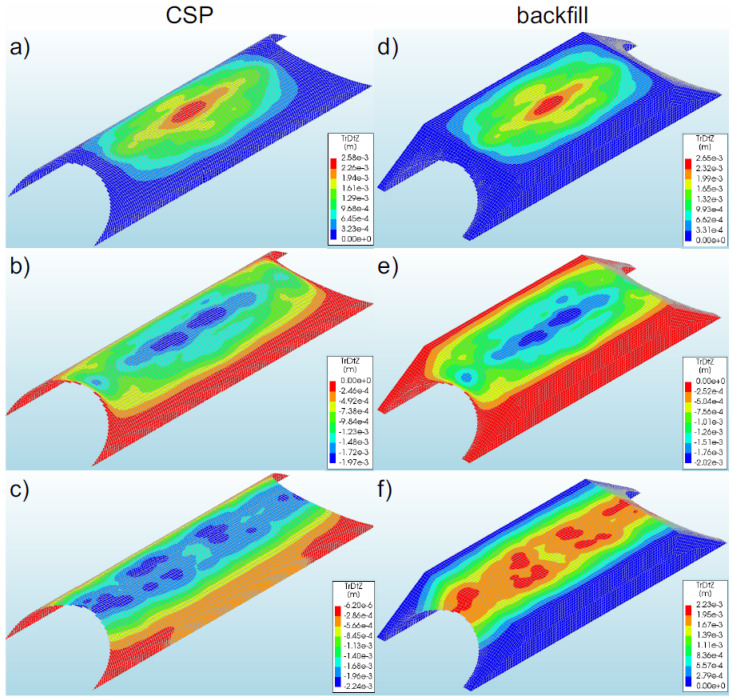
Maximum vertical displacements of the shell and backfill for bridge #I in model 1 (**a**,**d**), model 2 (**b**,**e**), and model 3 (**c**,**f**).

In general, the displacements of bridge #1 subjected to the earthquake were at a low level compared to the displacements of the shell obtained during backfilling process [6,10,12]. In the numerical analysis, the greatest displacements were recorded in model 1 (2.58 × 10^−3^ m—Figure 12a). In the remaining models, smaller values of displacement (in the range of 13–24% of the maximum value) were obtained, which amounted to −1.97 × 10^−3^ m model 2—Figure 12b) and −2.24 × 10^−3^ m (model 3—Figure 12c). A different direction of displacement of the steel shell in individual bridge models should be noted. In model 1, the steel shell was displaced upwards, whereas in models 2 and 3 it was displaced downwards.

The maximum displacement of the steel shell (model 1) was recorded in the region of the shell crown at half the length of the soil–steel composite bridge (Figure 12a). In model 2, maximum displacements occurred in similar places as in the case of model 1. However, in model 3, the greatest displacements were recorded in a larger area of the steel shell (Figure 12c). They were registered in the vicinity of the crown along the entire length of the top of the shell. It can be added that the use of the reinforcement RC collar applied to support the crown and reinforcement of the slopes of the model results in the concentration of the shell displacements in the middle of its length (model 1—Figure 12a). On the other hand, along with the reduction of the inlet and outlet stiffness in models 2 (only the shell reinforcement exists at the inlet and outlet of the bridge) and 3 (non-reinforced inlet and outlet), the range of vertical displacements increased. The largest range of displacements in the analysed models was recorded in model 3 (Figure 12c). It should be added that the greatest horizontal displacements (in X-direction) occurred in model 2, and the maximum value was −4.10 × 10^−3^ m.

Generally, the vertical displacements of the backfill were at a very similar level to those of the shell. Identical characteristics were also observed in the distribution of displacements in the backfill and in the shell (Figure 12). The highest value of backfill displacements was recorded in model 1 (2.65 × 10^−3^ m—Figure 12d). The displacements of the backfill were located in its upper parts, which may affect the safety and comfort of the road located on the site. However, in this case, the reported displacements of the backfill as a result of the earthquake were within the permissible level of deviations that were rendered acceptable in roads and their surfaces [55]. 

It can also be noted here that a difference of displacements was observed at the interface between the steel shell and the backfill. In model 2 (actual one), the maximum displacements in the interface layer amounted to −5.23 × 10^−4^ m and were smaller by about 73% than the displacements of the shell and the backfill.

#### 3.2.2. Bridge #II

**Figure 13:**
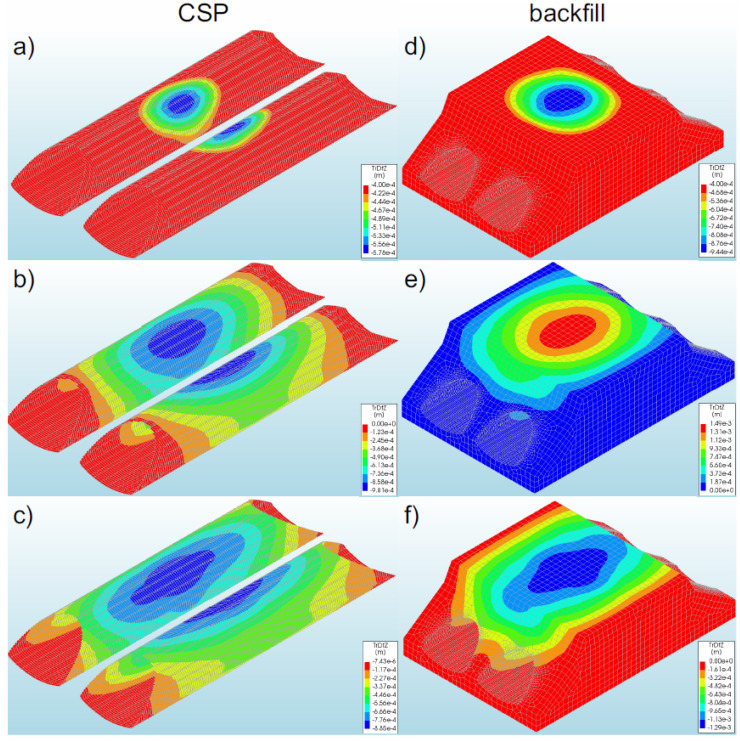
Distribution of the maximum vertical displacements for bridge #II in the steel shell and backfill for model 4 (**a**,**d**), model 5 (**b**,**e**), and model 6 (**c**,**f**).

In the analysed bridge #II, the greatest vertical displacements were recorded in model 5 (−9.81 × 10^−4^ m—Figure 13b). The study also discovered that the displacements in the remaining models were smaller, i.e., in the range from 10% to 41% in relation to the maximum values recorded for model 5. For models 4 and 6, the displacements were equal to −5.78 × 10^−4^ m (Figure 13a) and −8.85 × 10^−4^ m (Figure 13c), respectively. As a result of the application of the El Centro excitation, the upper parts of the soil–steel composite bridge shell were displaced downwards. The maximum displacements for the steel shell (in model 5) were observed in its crown, in the half of the length of the soil–steel bridge (Figure 13b). In the remaining models, the largest displacements were observed in the same locations as in the case of model 5, but these displacements differed only in the range of their occurrence.

The greatest range of the displacement was recorded in model 6 (Figure 13c). A positive effect of the RC collars and end front walls (models 4 and 5) on the distribution of vertical displacements in the steel shell and in the backfill was observed. It can also be added that the maximum horizontal displacements (in X-direction) were smaller than the vertical displacements and occurred in model 6, where their value was −4.44 × 10^−4^ m. 

Generally, the vertical displacements of the backfill assumed very similar levels as the vertical displacements of the shell. The maximum value was obtained in model 5 (Figure 13e), which was 1.49 × 10^−4^ m. A similar tendency was also observed in the distribution of displacements in the backfill and in the shell (Figure 13). The only differences were in the range of the maximum values. Backfill displacements occurred in the upper parts of the model. In addition, the recorded track displacements were within the permissible deviations specified for this type of structure [55].

As a result of the seismic excitation, it was noticed that there were small displacements in the interface layer (at the junction of the steel shell and the backfill), the maximum value of which was −2.16 × 10^−4^ m (model 5). Thus, they were definitely smaller than the displacements of the shell (by 78%) and the backfill (by 86%).

### 3.3. Stresses

#### 3.3.1. Bridge #I

**Figure 14:**
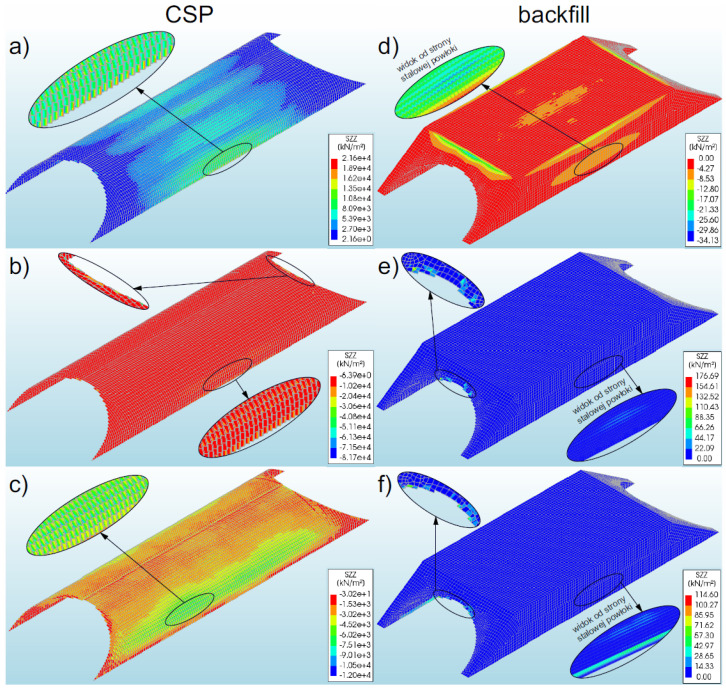
View of the maximum global stresses (in Z direction) for bridge #I in the steel shell and backfill for model 1 (**a**,**d**), model 2 (**b**,**e**), and model 3 (**c**,**f**).

The study discovered that the highest global stresses (in Z direction) occurred in model 2 and assumed the value of −81.7 MPa (Figure 14b). In the remaining models, lower stresses were obtained (in the range of 74–85% of the maximum value). In model 1, the recorded maximum global stresses were equal to 21.6 MPa (Figure 14a), whereas in model 3 the value was −12.0 MPa (Figure 14c). In model 2, the maximum global stresses were recorded at the inlet and outlet of the bridge, and in models 1 and 3, they occurred in the lower parts of the numerical model (i.e., at the foundation). It can be emphasized that in all models of the soil–steel composite bridge, the maximum global stresses in the shell occurred locally. The most obvious local characteristics of the occurrence of the greatest global stresses in the shell can be indicated in model 2, where the maximum value occurred only locally (Figure 14b). In addition, a slight increase in stresses in model 2 was recorded near the foundation, in similar places as the maximum stresses in models 1 and 3. The global stresses in this place (model 2, Figure 14b) were lower by about 75% compared to the maximum global stresses in this model. In other places of the shell, the stresses did not exceed 1% of the maximum values. The observed global stresses had uniform distribution all over the majority part of the shell surface. 

The global stresses in the backfill assumed a very low level and did not exceed 0.177 MPa (model 2, Figure 14e). In model 3 (Figure 14f), the maximum global stresses were recorded at the inlet and outlet of the bridge. In model 1, however, the maximum global stresses in the backfill occurred in the lower sections of the numerical model, above the maximum global stresses in the shell. It should be emphasized that the maximum global stresses in the backfill in all analysed bridge models occurred only locally. In other places, the stresses did not exceed a few percent of the maximum values.

#### 3.3.2. Bridge #II

**Figure 15:**
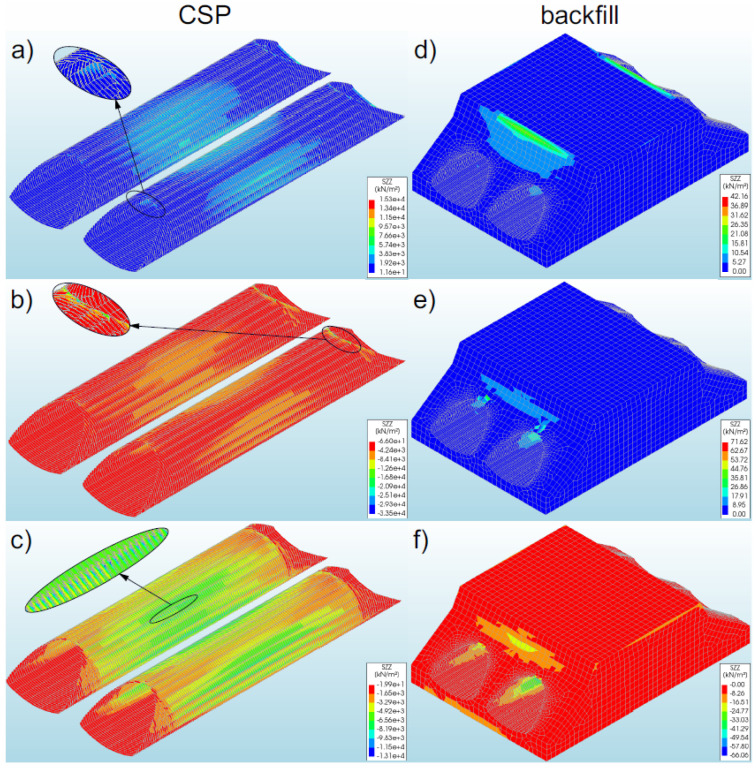
View of the maximum global stresses (in Z direction) for bridge #II in the steel shell and backfill for model 4 (**a**,**d**), model 5 (**b**,**e**), and model 6 (**c**,**f**).

The highest recorded global stress in the shell occurred in model 5 and was equal to −33.5 MPa (Figure 15b). In the remaining models (15.3 MPa—model 4, −13.1 MPa—model 6), lower global stresses were recorded in the range of 54–61% of the results compared to model 5. In models 4 and 5, the maximum global stresses were recorded at the inlet and outlet of the bridge (near the steel shell crown, Figure 15a,b). However, in model 6 they appeared in the middle of the length of the numerical model (Figure 15c), at quarter points (from the side of the adjacent shell). 

It should be emphasized that in all numerical models of a soil–steel composite bridge, the maximum global stresses in the shell occurred locally. This was particularly discernible for the case of models 4 and 5, in which the maximum results were recorded along a very small region around the shell (Figure 15a,b). In these models (4 and 5), an increase in stresses were recorded in similar places as in the case of model 6. However, the stresses were lower, i.e., in the range of 60–75% in comparison with the model 6. In other places, the obtained results did not exceed a few percent of the maximum values. In most areas of the shell, the global stresses observed were much lower and their distribution was regular. Moreover, it was noticed that the stresses in the backfill were at a much lower level than in the shell and did not exceed 0.071 MPa (model 5), as demonstrated graphically in Figure 15e. It should be noted that the locations marked by the highest global stresses in the analysed models were the same as in model 6, i.e., in the middle of their length (at the quarter points of the shells). 

### 3.4. Axial Forces

#### 3.4.1. Bridge #I

**Figure 16:**
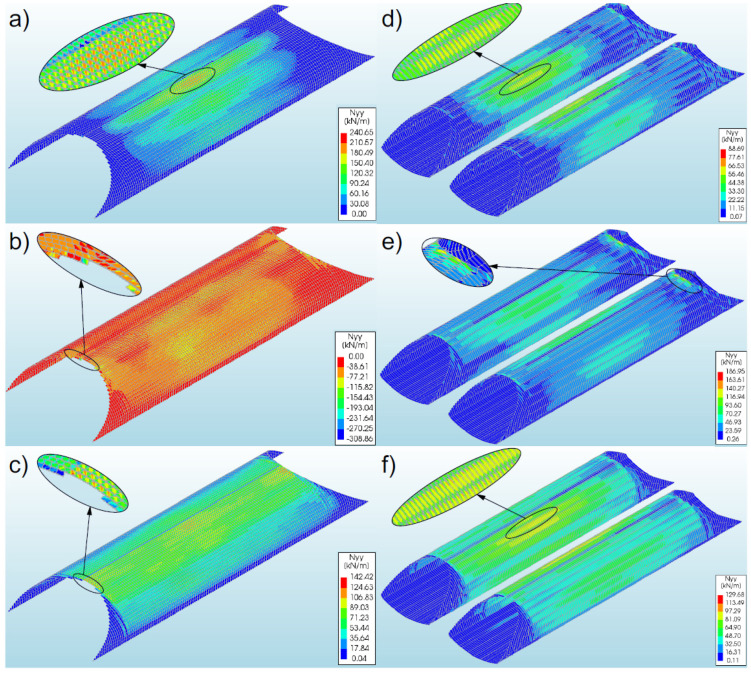
Distribution of maximum axial forces in the steel shell for models (**a**) 1, (**b**) 2, and (**c**) 3 (bridge #I); and for models (**d**) 4, (**e**) 5, and (**f**) 6 (bridge #II).

The greatest axial forces (−308.86 kN/m) occurred in model 2. They were recorded in the steel shell crown, at the inlet and outlet sides (Figure 16b), and they assumed negative values (compression). In the remaining models of the bridge, the axial forces were lower. In models 1 and 3, the values were 240.65 kN/m and 142.42 kN/m, respectively (i.e., smaller by 22% and 54% in comparison with model 2). The forces in these models assumed positive (tension) characteristics. In general, the maximum axial forces in models 1–3 were observed in the steel shell crown, but in model 1 they occurred in the middle of the bridge span (Figure 16a), and in models 2 and 3 they were observed at the inlet and outlet of the shell (Figure 16b,c). It should be noted that the maximum values of axial forces for individual models were recorded only locally. The locations of the occurrence of the maximum values corresponded to the extremes of these forces. In the remaining parts of the shell, the distribution of axial forces was uniform, and the values were approximately three to four times lower from the maximum ones. Moreover, it was noticed that the use of front walls (for slope reinforcement—model 1) positively affected the level of axial forces in the shell, compared to model 2, where the inlet and outlet stiffening was applied only on the shell side. Additionally, the end walls concentrate the axial forces in the middle of the shell. In models 2 and 3, an increase in axial forces was also recorded in the steel shell crown, in the middle of the shell length. The value of these forces ranged from 63–88% of the maximum value for these models.

#### 3.4.2. Bridge #II

The highest axial forces (186.95 kN/m) were recorded in model 5 and they assumed were positive values (tension). They were registered in a steel shell crown, from the inlet and outlet sides (Figure 16e). In models 4 and 6, the axial forces were lower and amounted to 88.69 kN/m (lower by 53% compared to model 5) and 129.68 kN/m (lower by 31%), respectively. These forces were recorded in the middle of the model length (Figure 16d,f), in the vicinity of the quarter points. When axial force maps are analysed (Figure 16e,f), it can be observed that the maximum values for individual models were obtained locally, and it was most discernible in the case of model 5 (Figure 16e), where the maximum axial forces were recorded on a very small surface of the shell. In the remaining parts of the shell, the distribution of axial forces was uniform, and the values were approximately three to four times lower than the maximum. After the numerical analysis, it was observed that as a result of the use of RC collars at the inlet and outlet (model 5), the axial forces concentrated in these locations and their value increased. On the other hand, in the central part of the shells, the axial forces were reduced by over 60%.

### 3.5. Bending Moments

#### 3.5.1. Bridge #I

**Figure 17:**
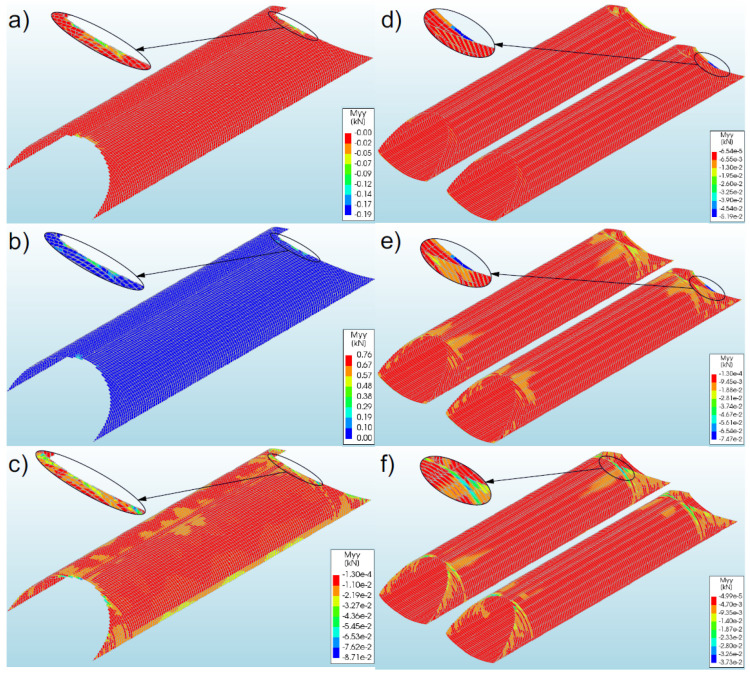
Distribution of maximum bending moments in the steel shell in models (**a**) 1, (**b**) 2, and (**c**) 3 (for bridge #I); and for models (**d**) 4, (**e**) 5, and (**f**) 6 (for bridge #II).

Generally, the numerical analysis of the soil–steel composite bridge demonstrated a very low level of bending moments in the corrugated steel shell for all the considered models (#1–3) with variable boundary conditions. The maximum bending moments (0.76 kNm/m) occurred in model 2 (Figure 17b) and they assumed positive values. In the remaining models, lower values were obtained. In the considered models (#1–3), the maximum bending moments were recorded at the ends of the steel shell (inlet and outlet), near the shell crown. It can be seen that the obtained maximum bending moments occurred locally, and their greatest value appeared only on a part of the finite elements. It was also noticed that the lack of the RC collar (model 3) resulted in the irregular distribution of moments in the shell. In addition, the use of a reinforced concrete collar or reinforcement of the model slopes leads to the concentration of bending moments in the vicinity of the bridge inlet and outlet (Figure 17a,b)

#### 3.5.2. Bridge #II

Just as in the case of a single-shell bridge, also in this case the bending moments were at a low level. The maximum recorded bending moments assumed the value of −0.075 kNm/m (Figure 17e) and occurred in model 5. They were recorded at the inlet and outlet of the soil–steel bridge, in the lower parts of the steel shell, and were negative. In the remaining models, lower values were obtained. In model 4, they were also observed at the ends of the steel shell (inlet and outlet). However, in model 6 (Figure 17f) it was recorded that the lack of the RC collar resulted in a shift of the maximum bending moments from the lower part of the shell to its crown (at the inlet and outlet). It can be concluded from the above that the recorded bending moments in the shells occurred locally, and the obtained results were extremes. In the remaining parts of the shells, the bending moments were very low or equal to zero.

### 3.6. Velocities

#### 3.6.1. Bridge #I

**Figure 18:**
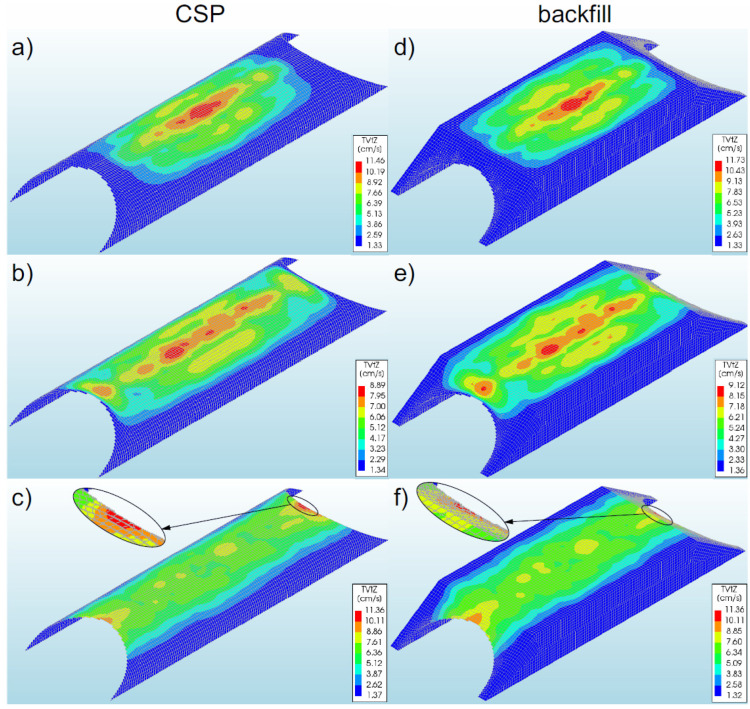
View of maximum velocities for bridge #I in the steel shell and backfill in model 1 (**a**,**d**), model 2 (**b**,**e**), and model 3 (**c**,**f**).

The highest velocities of the steel shell (11.46 cm/s—Figure 18a) and the backfill (11.73 cm/s—Figure 18d) were recorded in model 1. In the remaining numerical models analysed, lower values of the shell velocities were obtained (in the range of 1–22%). The maximum shell and backfill velocities (Figure 18a,b,d,e) in models 1 and 2 were observed in its shell crown, in the middle of the bridge span. It should be added that these values occurred locally. In contrast, in model 3, maximum values were also recorded in the shell crown, but at the inlet and outlet of the steel shell (Figure 18c). 

In general, it can be stated that the highest velocities occurred in the top sections of both the shell and the backfill. It can be remarked that significant shell and backfill velocities (for all models) occurred only from the half of the height of the shell cross-section and increased in the direction of the crown. In the lower sections of the shell (at the foundation), the shell and backfill velocities were very low (constituting a few percent of the maximum values recorded in a given model). In addition, it was observed that the use of a reinforcement collar as well as/or reinforcement of the bridge slopes affects the occurrence of the velocity both in the shell and in the backfill. Failure to use these reinforcing elements leads to the shift of the maximum velocities from the middle of the model (1 and 2) to the inlet and outlet (model 3).

#### 3.6.2. Bridge #II

**Figure 19:**
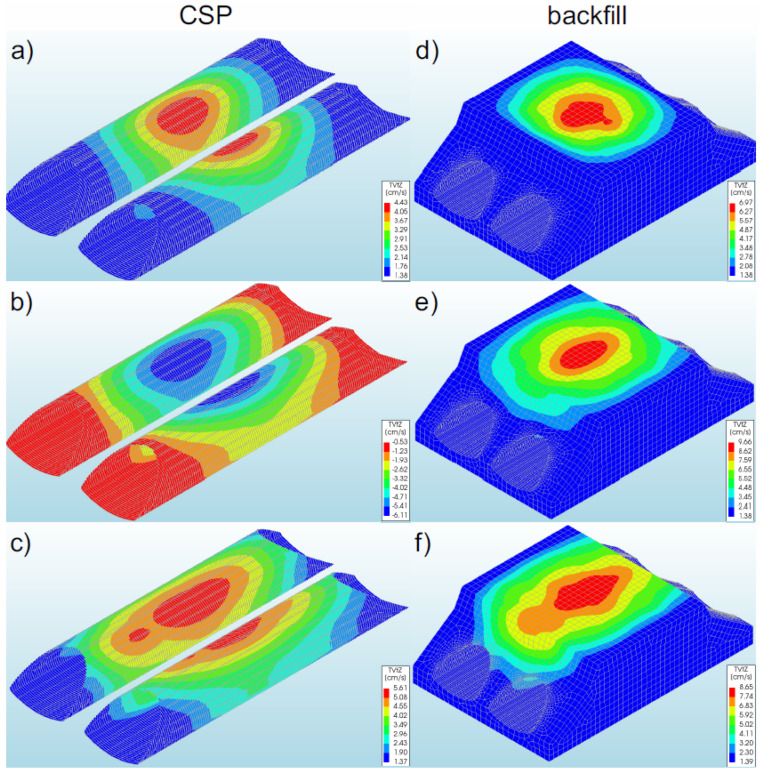
Maximum shell and backfill velocities for bridge #II in model 4 (**a**,**d**), model 5 (**b**,**e**), and model 6 (**c**,**f**).

The maximum velocities in the steel shell were recorded in model 5 and assumed a value of 6.11 cm/s (Figure 19b). In the remaining models, lower shell velocities were obtained in the range of 8–27% in comparison with results for model 5. The maximum shell velocities in the analysed models 4–6 were observed in the middle of the bridge span, in the shell crown (Figure 19a–c). It was noticed that these values occurred locally and concentrated in one point along the shell. 

From the maps presented in Figure 19, it can be seen that the significant shell velocities for all models became visible only from the middle of the height of the shell cross-section and they increased in the direction of the shell crown. In the lower part of the shell (at the base of the model), the shell velocities were very low (as they constituted a few percent of the maximum values recorded in a given model). In addition, it should be emphasized that the use of different boundary conditions increased the range of significant shell and backfill velocities. In model 4 (stiffened inlet and outlet of the shell with the slope), the velocities were concentrated in a smaller area of the shell and backfill. With the increase in model compliance (decrease in inlet and outlet stiffness, models 5 and 6), the range of maximum velocities was greater.

For the case of backfill, significant velocities (exceeding 2.0 cm/s) were observed only from the middle of the cross-sectional height of the numerical model for all models, as in the case of the steel shell and they increased towards the top part of the model (Figure 19). In addition, the location of the velocity in the backfill (upper part of the model, in the middle of its length) was also almost identical to that in the case of the shell.

### 3.7. Accelerations

#### 3.7.1. Bridge #I

**Figure 20:**
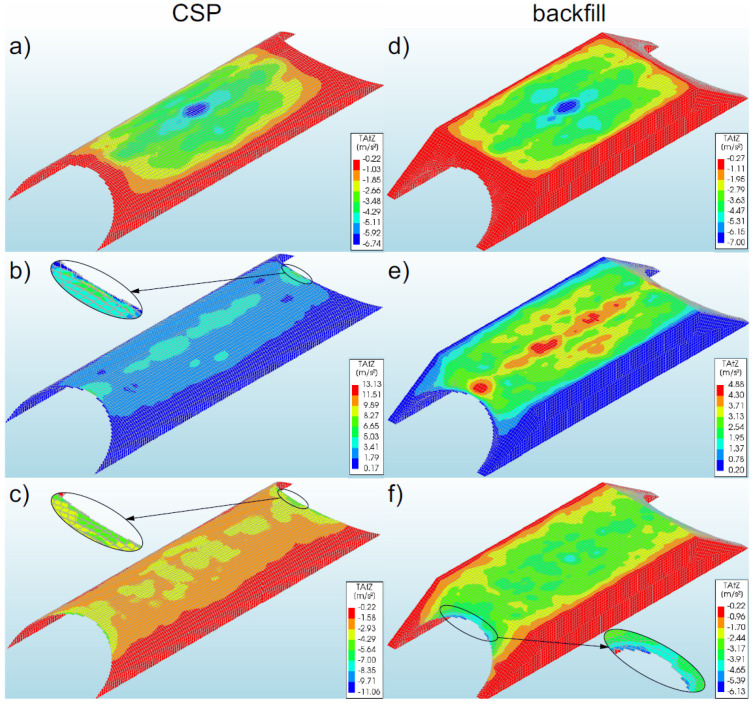
Distribution of maximum accelerations for bridge #I in the shell and backfill for model 1 (**a**,**d**), model 2 (**b**,**e**), and model 3 (**c**,**f**).

The maximum accelerations of the steel shell (13.13 m/s^2^) were recorded in model 2 (Figure 20b). In the remaining models, accelerations were lower, i.e., by 49% for model 1 (6.74 m/s^2^, Figure 20a) and by 16% (11.06 m/s^2^, Figure 20c) for model 3. However, the maximum accelerations of the backfill (7.0 m/s^2^) were recorded in model 1 (Figure 20d). In the remaining models (2 and 3), lower accelerations were obtained, i.e., in the range of 1–30%. 

In model 1, the maximum accelerations of the shell and backfill were obtained in the shell crown (in the middle of the span of the soil–steel bridge). In the remaining models (2 and 3), the greatest accelerations were located in different locations. In model 2, they were observed in the shell crown (inlet and outlet). However, in the backfill, they were also noted in the shell crown, but they were located along the entire length (upper part) of the numerical model (Figure 20e). In turn, in model 3, maximum accelerations both in the shell and in the backfill were obtained at the ends of the shell (inlet and outlet, Figure 20f).

The results recorded in the analysed models occurred only locally. This is best discernible in models 2 and 3, where the maximum values occurred only in a very small area of the shell. It can be added that significant accelerations for the analysed models 1–3 were obtained, similarly to the velocities, in the top parts of the shell. In the remaining area of the shell, accelerations did not exceed several percent of the maximum accelerations for a given model. In addition, the accelerations in the backfill were at a lower level than the accelerations of the steel shell. The results also indicated that the use of slope reinforcements (model 1) leads to the concentration of maximum accelerations in the middle part. In models 2 and 3, where no slope reinforcement was used, the maximum values moved to the inlet and outlet of the bridge models.

#### 3.7.2. Bridge #II

**Figure 21:**
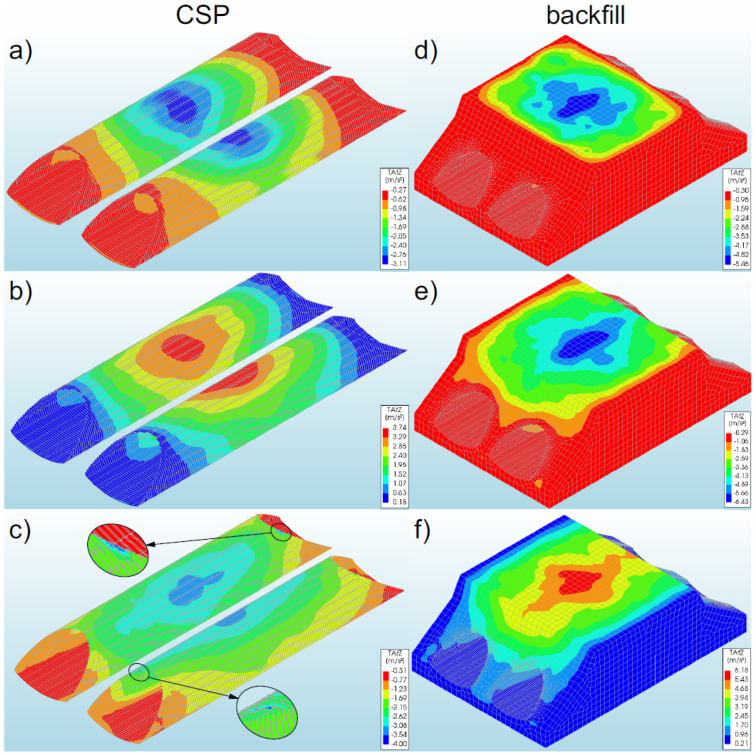
Maximum accelerations for bridge #II in the steel shell and backfill for model 4 (**a**,**d**), model 5 (**b**,**e**), and model 6 (**c**,**f**).

The highest values of accelerations in the corrugated steel shell were recorded locally in model 6 (Figure 21c), which amounted to 4.0 m/s^2^. In the remaining models, accelerations were lower, i.e., by 22% in model 4 (3.11 m/s^2^, Figure 21a) and by 7% in model 5 (3.74 m/s^2^, Figure 21b). In model 6, the maximum shell accelerations were recorded in its shell crown, from the inlet and outlet side of the soil–steel bridge. In the remaining models (4 and 5), the greatest accelerations were observed in the shell crown, in the middle of the span of the structure. It was noted that significant accelerations, similarly to velocities, were obtained in the upper sections of the shell. In the remaining area of the shell, the accelerations did not exceed several percent of the maximum values specified for a given model. In addition, it was observed that the tendency of the occurrence of accelerations of significant value was the same as in the case of velocities. The more flexible the inlet and outlet of the bridge was (less stiffened), the greater range of accelerations with higher values was observed (about 50% of the maximum value).

It can be noted that the greatest accelerations in the backfill occurred in the upper parts of the bridge model, above the shells (Figure 21d–f); in other areas, they were much lower. The maximum accelerations in the backfill (6.43 m/s^2^—Figure 21e) were higher than in the steel shell by an average of 56–76%.

## 4. Discussion

### 4.1. Influence of Boundary Conditions

**Figure 22:**
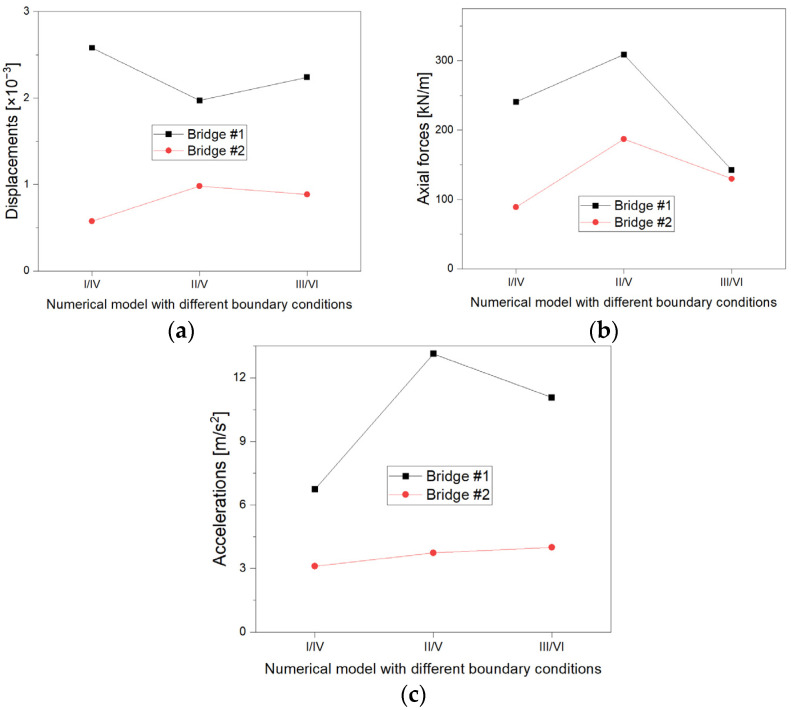
Comparison of displacements (**a**), axial forces (**b**), and accelerations (**c**) for bridge #I and #II.

The obtained results indicate certain trends in the impact of the application of particular boundary conditions in numerical models. Figure 22 shows that a change in the configuration of the boundary conditions (application or lack of reinforcements) has a greater impact on the response of bridge #1 to seismic load than bridge #2. This is undoubtedly related to the much larger span of bridge #1 than bridge #2 and its greater sensitivity to seismic loads. In addition, it can be seen that the lack of reinforcements (models III and VI) does not result in increased displacements, axial forces and accelerations compared to models with RC collars and RC front walls (#I-II, IV-V). It was also noticed (Figure 12, Figure 13, Figure 20 and Figure 21) that too much stiffening of the analysed bridges (models I and IV) causes concentrations of displacements and accelerations in the middle part of the shell and backfill. On the other hand, the lack of any reinforcements means that the end parts of the shell (inlet and outlet) are exposed to excessive deformation due to an earthquake. It follows that structural solutions should be sought to strengthen this type of bridges, which on the one hand will protect the ends of the shell, but at the same time do not increase the stiffness of the entire bridge too much. Therefore, as the analysis shows, the use of models II and V (with RC collars only) seems to be the optimal solution in this case. 

In addition, some correlations can be noticed, which may be caused by the given boundary conditions. Based on the displacements (Figure 22a), it can be seen that the values between models II and V (real bridges) were the most similar. It can be seen that such results may be influenced primarily by the span of the shell, while the application of individual conditions has a lesser impact on the obtained results. On the other hand, the spans are about 18 m and 4 m for the bridges #I and #II, respectively, so the disproportion was about four times. In turn, a similar difference in the obtained results (about four times) was observed only in the case of models I and IV. This leads to the conclusion that the smaller stiffening at the inlet and outlet (slope and RC collar) causes the blurring of differences in the response of bridges for the individual tested boundary conditions and for their different spans.

A similar tendency can be observed in the case of axial forces (Figure 22b), where the differences in the values of axial forces in particular types of numerical models with different boundary conditions disappear with the greater flexibility of the steel shell (inlet and outlet less rigid). On the other hand, in the case of maximum accelerations (Figure 22c), a rather opposite trend can be observed than in the case of displacements and axial forces, because with the increase in the flexibility of the shell, the differences between bridge #I and #II increase. This is logical, because the inlet and outlet, having more freedom, can achieve greater acceleration (not constrained by boundary conditions). 

### 4.2. Displacement of CSP Shell over Time

In seismic analysis, the duration of the seismic record is very important. In addition, an equally important aspect during seismic analysis is the behaviour of the object over time. In practice, such information is difficult to gain, because to obtain it, the structure would have to be constantly monitored. Unfortunately, this approach is expensive, and therefore its popularity is limited. In the case of soil–steel bridges, selected points used to observe the behaviour of the shell during its backfilling and normal service are analysed. Therefore, based on research [12] for bridge #1 and [28,32] for bridge #2, it was decided that the main parameter in these studies (analysis of the behaviour of the structure in time) would be displacements in the bridge crown, which is why this part of the work analyses displacements in time for bridge #I and #II. This analysis was carried out for models of real objects, i.e., models II and V. These values are important because by the maximum displacement it is possible to determine whether the object is suitable for use or not [55]. 

Previous studies [51] showed that the effect of the length of the seismic record is secondary. The duration of the intensive zone with maximum acceleration is more important than the duration of the entire record. The research showed that the El Centro record is the most unfavourable for the tested structure.

**Figure 23:**
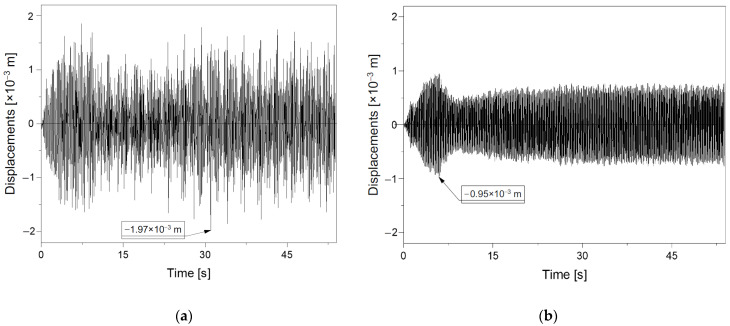
Maximum displacements in time of earthquake excitation for bridge crown: (**a**) #I—model II and (**b**) #II—model V.

Based on Figure 23, it can be seen that in both cases the maximum displacements were recorded during the intense phase of the El Centro recording (Figure 11). This zone lasted until about 30 s of recording, which is very long for a seismic shock. In the case of bridge #I, the displacement values (Figure 23a) were at a similar level throughout the recording process. On the other hand, in the case of bridge #II, it can be seen that with the beginning of the El Centro intensive recording zone (Figure 23b), a significant excitation is visible, where the maximum value was obtained. 

It can be concluded that the analysis over time is important for the response of the structure to a seismic shock. This behaviour shows how the structure copes over time with such a heavy load as an earthquake (e.g., El Centro).

### 4.3. Standard Approach

In general, design standards for determining the effects of seismic tremors on soil–steel objects have not been developed so far. The only standard in which this issue is addressed to a small extent is the Canadian standard (CHBDC, Canadian Highway Bridge Design Code [56]). The approach specified in this standard is very conservative, as it ignores the effect of intensity and duration of seismic excitation and focuses on the maximum acceleration of the ground. However, designing in accordance with the CHBDC, a significantly overdesigned steel shell is obtained. In addition, this standard defines the seismic effect based only on the simple multiplication of the axial force by a factor. Therefore, in such a case, there can be no precise determination of seismic influences on soil–steel bridges. Eurocode [50] and AASHTO [57] do not contain the specific requirements for designing the soil–steel bridges subjected to seismic excitations. It should be mentioned that the boundary conditions are very important, however these issues are ignored in the standards. As demonstrated in the analysis of numerical models, the differences in the results obtained can be significant [35]. 

Moreover, deformations of structure are very important in other regulations ( these cases were surface of roads and rail tracks). After numerical analysis, the most significant deformations recorded in the backfill in bridge #I and #II were located in the upper sections of the models and were within the permissible deviations that should be met by roads [55] and rail tracks [58]. Nevertheless, designers should take into account that in some cases larger deformations may occur, which may affect the comfort and safety of road and railway use.

## 5. Conclusions

On the basis of the numerical analysis, it can be concluded that the boundary conditions (constituting various structural reinforcements (RC collars, RC front walls)) located on individual edges of the numerical model of soil–steel composite bridges have a significant impact on the behaviour of these structures under strong seismic excitation. A similar tendency was observed in the behaviour of both analysed bridges. The most important detailed conclusions derived from the analysis include:

- the use of slope stiffening and inlet and outlet stiffeners (models 1 and 4) leads to a decrease in the maximum values in the shell (e.g., displacements or stresses) compared to models (2 and 5), where only the inlet and outlet of the shell itself were reinforced;
- the use of the RC collars acting as stiffening to the inlet and outlet of the shell and stiffening the slopes (models 1 and 4) leads to the concentration of displacements and velocities in the middle part of the shell;
- the use of stiffening in the form of RC collars and front walls (models 1 and 2) contributes to more uniform distribution of accelerations in the shell and backfill;
- the use of the RC collars from the inlet and outlet sides of the soil–steel composite bridge (without stiffening of the slopes, models 2 and 5) offers an increase in maximum stresses and axial forces in the shell and stress concentration at the ends of the shell;
- lack of stiffening at inlet and outlet (models 3 and 6) increases the region occupied by significant values (displacements, velocities, accelerations) and increases the values in relation to models 1 and 4 (stiffening at inlet and outlet of the shell and slope);
- the impact of boundary conditions on the bending moments in the shell was negligible. In all analysed models (1–6), the maximum bending moments occurred on the inlet and outlet sides, and the obtained values were low;
- in the case of displacements and axial forces, along with the increase in the flexibility of the shell in the numerical model (models III and VI), smaller differences between the boundary conditions for the tested bridges (#I and #II) were visible;
- from the practical point of view, the use of stiffening such as RC collars (at the inlet and outlet of the shell) and RC front walls (on the bridge slope) leads to a more regular distribution of the maximum values recorded in numerical models;
- the most significant deformations recorded in the backfill in bridge #I and #II were located in the upper sections of the models and were within the permissible deviations that should be met by roads [54] and rail tracks [58]. Nevertheless, designers should take into account that in some cases larger deformations may occur, which may affect the comfort and safety of road and railway use.

In addition, the conducted numerical analysis of soil–steel bridges with different boundary conditions may allow designers to select appropriate calculation assumptions to assess the behaviour of such structures under seismic excitation. 

It can be emphasized at this point that the analysed soil–steel composite bridges were investigated as new structures; free of any damage, subjected to adequate backfill compaction, and with no signs of corrosion. In an actual facility, in particular one that was operating for some time, there is a risk that the backfill will be characterized by variable degrees of backfill compaction, and the steel shell may have traces of corrosion resulting from water flow or mechanical damage [59]. These aspects undoubtedly affect the durability and safety of this type of bridges and may affect their response during an earthquake.

In summary, more extensive research is needed on soil–steel composite bridges exposed to earthquakes, in particular ones that already undergo some kind of damage (corrosion, loose connections). In the future, further research (numerical and experimental) will be undertaken on the dynamic identification of soil–steel composite bridges. In particular, the amount of damping by the backfill depending on the type of soil, the behaviour of the contact layer, the impact of relieving and damping elements, and a more in-depth analysis of the elements stiffening soil–steel bridges will be investigated. An important aspect that is also planned to be addressed will be the use of seismic loads of various intensity and duration. In addition, the authors plan to perform an experiment on a shaking table, which will allow experimental verification of numerical calculations. 

## Figures and Tables

**Table 1:** Material properties of soil–steel composite bridges.

Properties of Materials		
	Bridge #I	Bridge #II
Corrugated steel plate shell		
Young modulus (GPa)	205	
Density (kg/m^3^)	7850	
Poisson ratio	0.3	
Strength yield (MPa)	235	
Backfill		
Soil model	Nonlinear Duncan-Chang	
Density (kg/m^3^)	2050	
Young modulus (MPa)	100	
Poisson ratio	0.2	
Friction angle (^o^)	39	
Dilatation angle (^o^)	5	
Cohesion (kPa)	3.0	
Failure ratio	0.7	
Unloading-reloading stiffness (N/m^2^)	1000	
Reference pressure (N/m^2^)	101,350	
Unloading–reloading curve	0.25	
Backbone curve	1.1	
Minimum compressive stress	350	
Minimum tangential stiffness of the backbone curve	1200	

## Data Availability

Not applicable.
